# The distribution of seaweed forms and foundational assumptions in seaweed biology

**DOI:** 10.1038/s41598-024-73857-z

**Published:** 2024-09-28

**Authors:** João P. G. Machado, Vinícius P. Oliveira

**Affiliations:** 1 Institute of Biology, Federal University of Rio de Janeiro (UFRJ), Rio de Janeiro, Brazil; 2 Institute of Biology, State University of Rio de Janeiro (UERJ), Rio de Janeiro, Brazil

**Keywords:** Functional diversity, Functional traits, Macroalgae, Morpho-functional, Trait-based ecology, Ecology, Ecophysiology, Evolutionary ecology, Evolution, Marine biology

## Abstract

Seaweeds are the most phylogenetically diverse group of multicellular organisms and rank foremost among marine keystone species. Due to their taxonomic diversity and functional importance, previous studies have classified seaweeds into functional groups based on qualitative or semi-quantitative traits, such as seaweed form, anatomy, and thickness. Despite the widespread use of seaweed functional groups from basic marine ecology to coastal monitoring, it is not known how accurate such morphology-based proposals are in grouping seaweeds by their form. To address this uncertainty at the foundations of seaweed biology, we surveyed and gathered all available data on seaweed forms using PRISMA protocols. We used the surface area to volume ratio (SA:V), a quantitative and universal measure of seaweed form, to assess the distribution and diversity of seaweed morphology across 99 species from three phyla. We show that seaweed surface area to volume ratio values span 3.64 orders of magnitude and follow a continuous and exponential distribution, without any significant gaps or clusters. We also tested current functional group schemes based on morphology and anatomy and showed that only 30% to 38% of their groups showed any significant pairwise differences in morphology. Our results challenge the basis of the current functional group approach in seaweed biology and suggest that a trait-based framework based on quantitative and continuous measures of seaweed form could provide a simpler and more accurate alternative to functionally assess seaweed ecology and physiology, as well as its implications for coastal ecosystem management.

## Introduction

Seaweeds are the most phylogenetically diverse group of multicellular organisms to ever exist on Earth^1,2^. They also play a vital role in the structure and functioning of marine ecosystems. Seaweed form and function have been a topic of interest since ancient times^3^ and the dawn of phycology^4^, but the relationship between seaweed morphology and functional traits is still debated^5,6^. Seaweed morphology influences many aspects of their biology, such as nutrient uptake, growth rate, photosynthesis, and interactions with other organisms. However, how can seaweed morphology be measured and compared across different species? Morphological traits are difficult to quantify and standardize among different taxa.

While various methods have been used to classify seaweeds based on their morphology^6–10^, the issue of cross-taxa applicability remains. Seaweed form takes a vast array of diversity which renders categorical approaches problematic and exclusive. To surmount this issue, the surface area to volume ratio (SA:V), measuring how much surface area is available per unit of volume, has been used as a quantitative alternative^9^. This ratio can vary widely among seaweeds, from thin and flat forms with a high surface area to volume ratio to thick and compact forms with a low surface area to volume ratio. The range of possible SA:V values for seaweeds defines their morphospace, i.e. the geometrical space of possible morphological variation^11–13^.

The surface area to volume ratio has an important relationship with other functional traits of seaweeds: nutrient uptake^14–17^; growth rates^18,19^; susceptibility to herbivory^20^; respiration and photosynthesis rates, as demonstrated by several studies^17,21–28^. It also has a negative correlation with nutrient storage^29^. These relationships stem from the ability of SA:V to map the relative quantity of photosynthetically-active tissues, determined mostly by thallus surface area, against that of structural tissues, determined mostly by thallus volume^9^.

Due to the relations between seaweed form and function, seaweeds have been grouped into morphology-based groups that correlate to similar functional traits and ecological roles^30–32^. These groups are based on the assumption that they hold greater within-group trait similarities than between groups, what has been deemed the “group gambit”^6^. Functional groups are a useful analytical tool to simplify and ordinate seaweed assemblages and to predict community processes^23,33–38^. Such groups are widely used in marine conservation and research, especially for their easiness of use by non-specialists instead of taxonomic screening^30^.

Different functional group schemes have been proposed, with different aims and classification criteria as we have recently reviewed^39^. For example, Littler and Littler^34^ classified seaweeds into six functional groups: “Crustose”, “Jointed Calcareous”, “Filamentous”, “Sheet”, “Coarsely Branched”, and “Thick and Leathery” based mostly on thallus thickness and degree of calcification. Steneck and Dethier^38^ proposed seven functional groups: “Crustose”, “Articulated Calcareous”, “Filamentous”, “Foliose”, “Corticated Foliose”, “Corticated Macrophyte”, and “Leathery Macrophyte”, based on the axis of cell division and thalli size. These two functional group schemes are the most cited to date and have been widely used in ecological studies of seaweed communities, especially in coral reefs^35,40–42^.

The use of categorical traits in functional groups is currently being reassessed^7,8,10,30^, but to date, it is not known whether the key assumption of seaweed functional groups is valid, which is that the seaweed morphospace is composed of discrete clusters (groups) rather than a continuous distribution. The nature of this distribution would then either (a) warrant categorical discrete treatment and support the functional group approach if discrete clusters were found or (b) warrant numerical continuous treatment and undermine the functional group approach if a continuous distribution was found.

Seaweed functional ecology has assumed that seaweed morphology has a rather clustered and discrete distribution, but these assumptions never were comprehensively tested^22,30,38,43–45^. Therefore, this paper poses and answers several questions about the foundations of seaweed ecology: How is the seaweed morphospace distributed? How does form vary between species, genera, and phyla? Is the functional group approach to seaweed form accurate? How quantitatively different are the most cited functional group models?

## Materials and methods

To answer this question, we conducted a comprehensive literature search on Web of Science, using the keywords ((“SA:V” OR ((“surface area” OR “superficial area”) AND “volume”)) AND (“seaweed” OR “macroalgae”)) using the standard PRISMA protocol^46^ (Supplementary Materials 1–3).

We extracted the metadata and surface area to volume values for 99 seaweed species covering a wide range of taxonomic and geographic diversity. We then plotted the surface area to volume ratio values by species, genera and phyla. Inter- and intra- specific, generic and phyletic differences were statistically assessed. For differences among phyla, Kruskal–Wallis test and Whitney U tests with Bonferroni correction were used. We calculated intrageneric and intergeneric variances using the raw and log-transformed SA:V data. Intrageneric variance was determined by calculating the variance of SA:V within each genus, followed by the variance of these intrageneric variances. Intergeneric variance was computed by calculating the variance of the mean SA:V for each genus. Similarly, intraspecific variance was determined by calculating the variance of SA:V within each species, and interspecific variance was derived from the variance of the mean SA:V for each species. F-tests were performed to compare intrageneric vs. intergeneric and intraspecific vs. interspecific variances. Levene’s test was used to assess the homogeneity of variances among genera and species. Additionally, the Kruskal–Wallis test was applied to evaluate differences in SA:V among genera and species, providing a non-parametric comparison of variances.

We also classified each species according to the two most cited functional group models: Littler and Littler model^34^ and Steneck and Dethier model^38^. We then plotted the surface area to volume ratio values per functional group and tested the significance of the differences between groups (Kruskal–Wallis, Dunn’s test). The difference in classification between the two proposals was also quantitatively assessed (Chi-squared, Jaccard similarity index).

## Results

### How is the seaweed morphospace distributed?

**Fig. 1:**
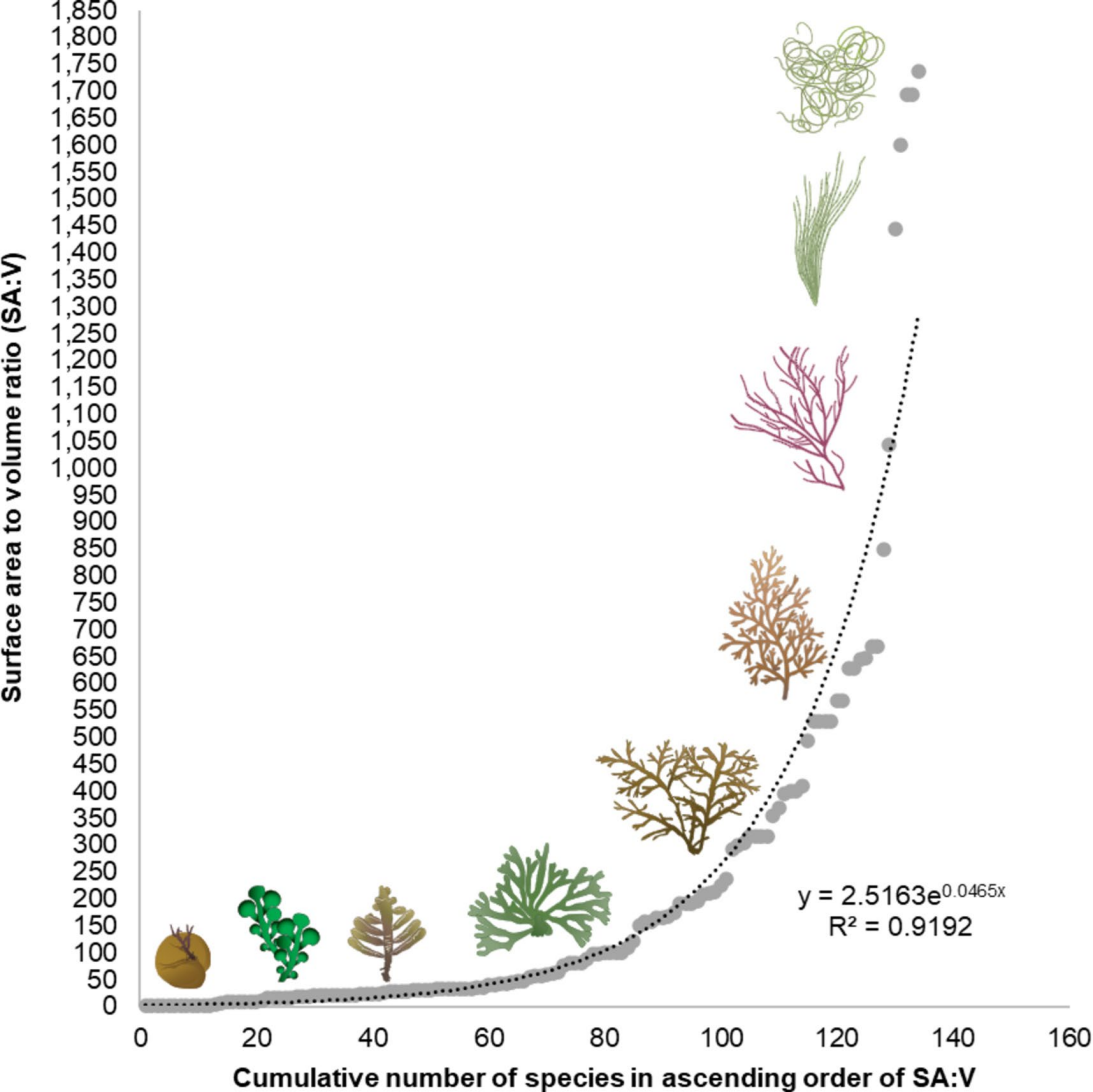
The seaweed morphospace by surface area to volume ratio. Illustrations represent SA:V gradation.

We obtained morphological data on 99 seaweed species, in 53 genera and three phyla, by surface area to volume ratio values as a quantitative metric for seaweed form assessment. The distribution of seaweed forms follows an exponential function (asymptotic one-sample Kolmogorov–Smirnov, D = 0.29179, *p* = 2.461 e^−10^ ; y = 2.5163 e^0.0465 x^ ; R^2^ = 0.9192). The surface area to volume ratio values exhibited a considerable range (0.53 to 1737), spanning 3.64 orders of magnitude, indicating extensive morphological diversity (Fig. 1).

### How does form vary between and within species, genera, and phyla?

**Fig. 2:**
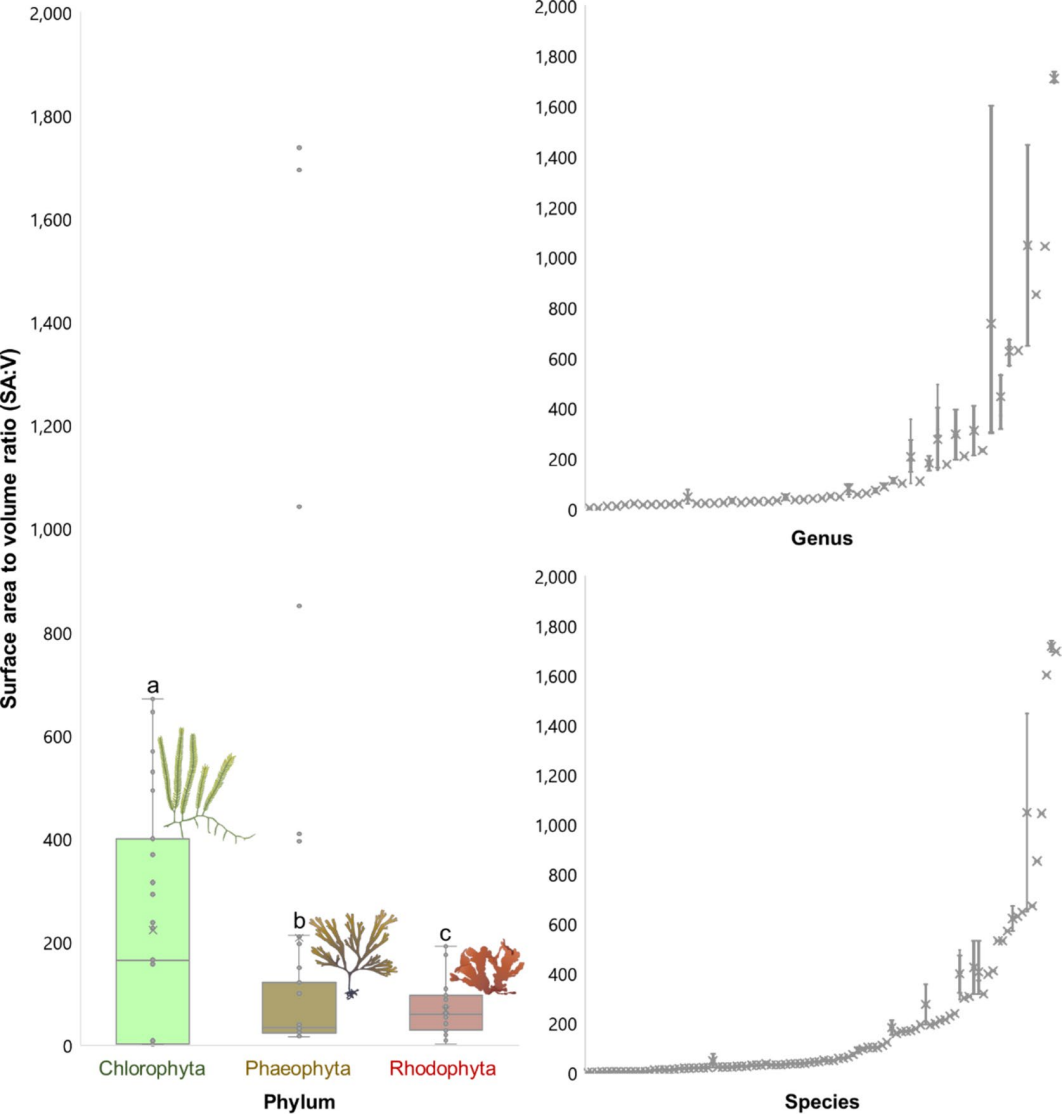
Surface area to volume ratio variation within and between seaweed phyla, genera and species. Taxon data are given as boxplots. Letters indicate significant differences.

Surface area to volume values showed significant differences while comparing the three phyla (Kruskal–Wallis, test statistic: 6.2878, *p* = 0.0431) (Fig. 2). Each phylum also showed significant differences when compared pairwise (Table 1). There were no significant variance differences among genera (Levene, F = 1.1487, *p* = 0.2842), only for species (Levene, F = 5.6475, *p* < 0.001). Significant SA:V differences were found among genera (Kruskal–Wallis, chi-squared = 129.74, *p* < 1.39 × e^−8^) and species (Kruskal–Wallis, chi-squared = 131.97, *p* = 0.01053). Intrageneric variance (7.75 × 10^15^, raw data; 0.0138, log-transformed) was lower than intergeneric variance (4.14 × 10^16^, raw data; 0.737, log-transformed) (F-ratio = 0.0187, raw data; F-ratio = 0.0188, log-transformed). Conversely, intraspecific variance (4.02 × 10^9^, raw data; 2.86, log-transformed) was higher than interspecific variance (1.19 × 10^5^, raw data; 0.31, log-transformed) (F-ratio = 9.26).

**Table 1:** Differences in surface area to volume between seaweed phyla.

Group 1	Group 2	U statistic	p-value	adjusted *p*-values
Rhodophyta	Phaeophyta	1074	0.0013	0.0039
Rhodophyta	Chlorophyta	1019	0.0036	0.0108
Phaeophyta	Chlorophyta	1100	0.0020	0.0060

### Is the functional group approach to seaweed form accurate?

**Fig. 3:**
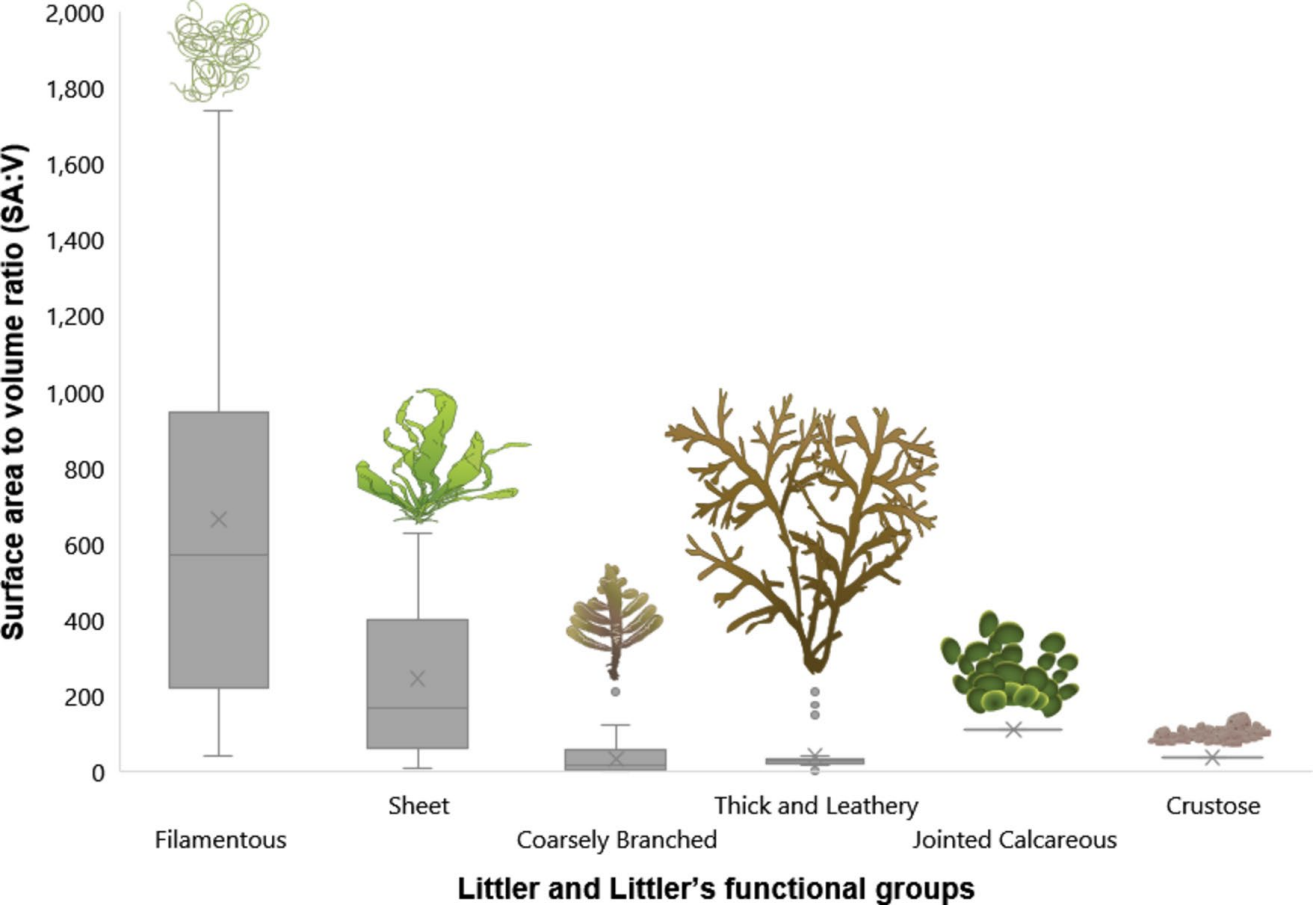
Surface area to volume ratio in Littler and Littler’s functional groups.

**Fig. 4:**
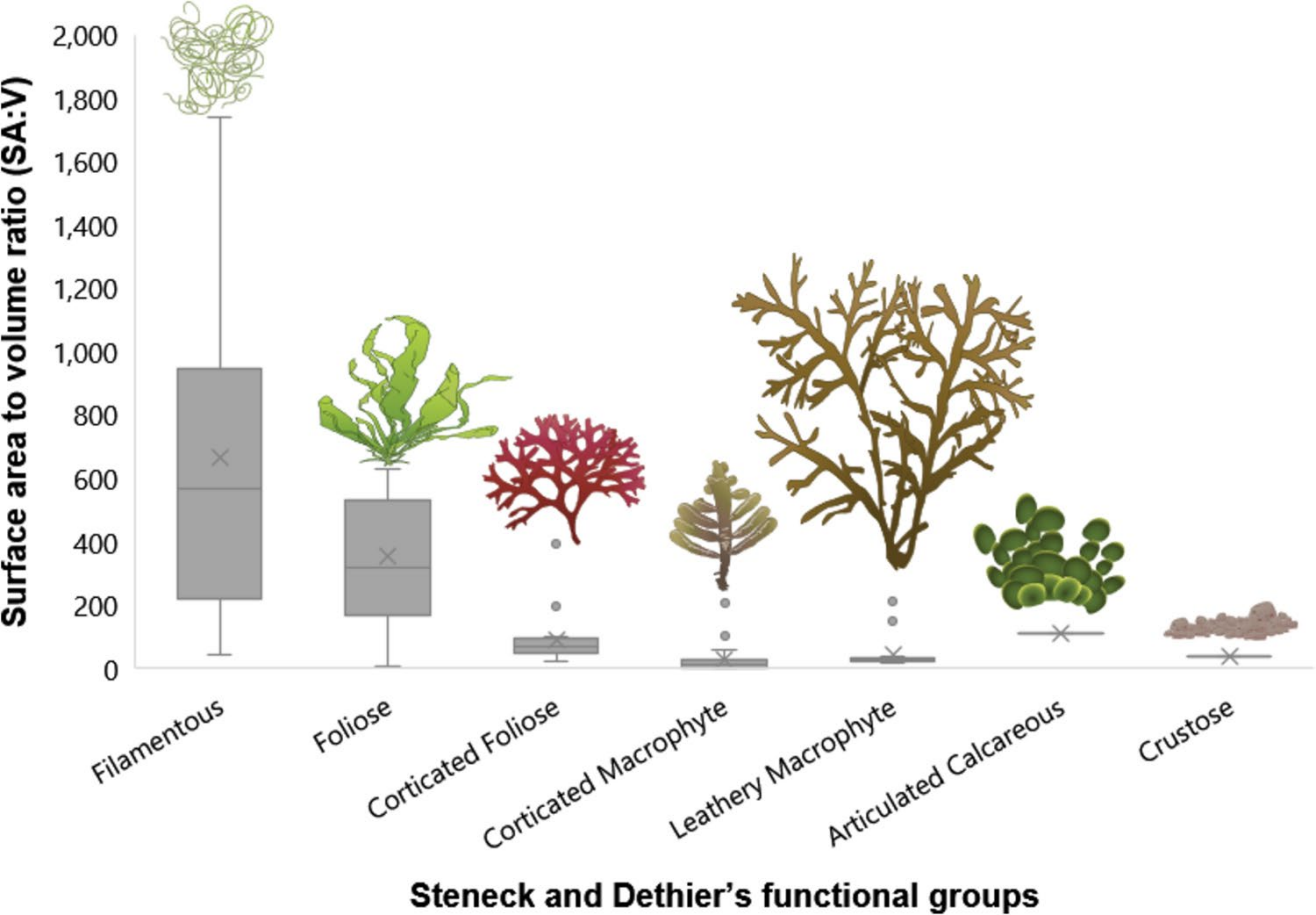
Surface area to volume ratio in Steneck and Dethier’s functional groups.

Using Littler and Littler^34^ functional groups (Fig. 3 and Table 2), only 3 out of 10 pairwise comparisons showed significant differences in morphology (Kruskal Wallis, *p* < 3.532 × e^−16^; Dunn’s test, *p*-values in Table 2). Using Steneck and Dethier^38^ functional groups (Fig. 4 and Table 3), only 8 out of 21 pairwise comparisons were significant (Kruskal Wallis, *p* < 2.2 × e^−16^; Dunn’s test, *p*-values in Table 3). Only 30% of Littler and Littler’s functional groups and 38% of Steneck and Dethier’s showed any significant pairwise differences in morphology.

**Table 2:** Pairwise comparison of morphological difference between Littler and Littler functional groups.

Functional group pairwise comparison	*p*-value	Significant
Coarsely branched vs. Jointed Calcareous	*p* = 1	No
Coarsely branched vs. Sheet	*p* = 1	No
Coarsely branched vs. Thick and Leathery	*p* = 1	No
Filamentous vs. Jointed Calcareous	*p* = 1	No
Filamentous vs. Thick and Leathery	*p* = 1	No
Jointed Calcareous vs. Thick and Leathery	*p* = 1	No
Jointed Calcareous vs. Sheet	*p* = 0.1717	No
Coarsely branched vs. Filamentous	*p* < 0.001	Yes
Filamentous vs. Sheet	*p* < 0.001	Yes
Sheet vs. Thick and Leathery	*p* < 0.001	Yes

**Table 3:** Pairwise comparison of morphological difference between Steneck and Dethier’s^38^ functional groups.

Functional group pairwise comparison	*p*-value	Significant
Articulated Calcareous vs Crustose	*p* = 1	No
Articulated Calcareous vs Corticated Foliose	*p* = 1	No
Articulated Calcareous vs Filamentous	*p* = 1	No
Articulated Calcareous vs Foliose	*p* = 1	No
Articulated Calcareous vs Leathery Macrophyte	*p* = 1	No
Crustose vs Corticated Foliose	*p* = 1	No
Crustose vs Foliose	*p* = 1	No
Corticated Foliose vs Corticated Macrophyte	*p* = 1	No
Corticated Macrophyte vs Foliose	*p* = 1	No
Filamentous vs Foliose	*p* = 1	No
Foliose vs Leathery Macrophyte	*p* = 1	No
Corticated Foliose vs Leathery Macrophyte	*p* = 0.351	No
Corticated Foliose vs Foliose	*p* = 0.135	No
Crustose vs Filamentous	*p* = 0.006	Yes
Crustose vs Corticated Macrophyte	*p* = 0.001	Yes
Articulated Calcareous vs Corticated Macrophyte	*p* < 0.001	Yes
Crustose vs Leathery Macrophyte	*p* < 0.001	Yes
Corticated Foliose vs Filamentous	*p* < 0.001	Yes
Corticated Macrophyte vs Filamentous	*p* < 0.001	Yes
Corticated Macrophyte vs Leathery Macrophyte	*p* < 0.001	Yes
Filamentous vs Leathery Macrophyte	*p* < 0.001	Yes

Kruskal Wallis, *p* < 2.2 × e^−16^; Dunn test *p*-values below.

### How quantitatively different are the most cited functional group models?

Classifications are significantly different overall (Pearson's chi-squared test, chi-squared = 644.08, df = 35, *p* < 2.2 × e^−16^). However, given that one proposal has 6 and the other 7 groups, an 85% similarity was mathematically expected upon classifying each species we surveyed. The Jaccard similarity index was very close to the expected (J = 0.8283582; J ≈ 83%).

## Discussion

### Finite forms most beautiful: the seaweed morphospace

Seaweeds have a great diversity of forms, resulting from a compromise between different evolutionary pressures scaling positively and negatively to surface area to volume ratios, such as nutrient uptake versus nutrient storage, productivity versus respiration, growth rate versus resistance to herbivory^14–29^.

While area scales by the square, volume increases by the cube. All else being equal, seaweeds of bigger sizes have lower surface area to volume ratio than smaller ones (e.g., *Chorda filum* and *Ectocarpus*). Spherical seaweeds have the lowest surface area to volume ratio than seaweed with other forms, such as fan-shaped ones (e.g., *Valonia* and *Udotea flabellum*).

Given that:

(*a*) The mathematical relationship between area and volume is exponential $(\frac{A}{V}=\frac{{\left[d\right]}^{2}}{{\left[d\right]}^{3}}={d}^{-1})$;

(*b*) productivity and respiration scale directly and inversely to surface area to volume ratio in seaweeds^17,21–28^;

(*c*) seaweeds need an overall positive net primary productivity^18,19^;

We expected the distribution of seaweed surface area to volume ratio data to be exponential. Nonetheless, it could conform to these requirements and have either unexplored morphospace regions along the curve or be continuous. If the distribution found had clusters or gaps, we would expect it to be discontinuous. This would entail having forms that although possible, had no extant species exploring that morphospace niche.

A continuous distribution in a morphospace without gaps should consist of an exponential curve f(SA:V) = *a* e^*b* x^, where *a* and *b* are empirical constants, and that is what we found for seaweeds from around the globe (f(SA:V) = 2.5163 e^0.0465 x^; R^2^ = 0.9192). This means that seaweeds currently explore the entire space of physically possible surface area to volume ratio and no seaweed ecomorphs or clusters of convergent morphologies exist, but they are significantly evenly distributed (asymptotic one-sample Kolmogorov–Smirnov, D = 0.29179, *p* = 2.461 × e^−10^). This results in the current seaweed functional group proposals being reductive regarding their morphological categories of seaweed groups.

The evenness in the seaweed morphospace is in stark contrast to the discrete occupation on the land plant morphospace^47,48^. Comparing the SA:V distribution with other trait-spaces, we noticed a similar trend towards an overall evenly dispersed occupation of general trait-space in seaweeds^6,7,10,49^. This fact when coupled with the billion-year antiquity of the “seaweed niche”^50^, leads us to speculate whether selection favored an optimum seaweed morphospace^13,51–53^ described by the function f(SA:V) = 2.5163 e^0.0465x^. Conversely, land plant evolution, roughly a third of the age of seaweeds^54^, together with perhaps more competing demands^49,55,56^, might have rendered their morphospace exploration more restricted, with the non-coincidence between the geometrically possible, physically possible, and operant morphospaces^13,57,58^.

### Inter- and intra- phyletic, generic, and specific variations in seaweed form

The analysis of surface area to volume ratios across different phyla revealed significant differences (Kruskal–Wallis, test statistic: 6.2878, *p* = 0.0431). Pairwise comparisons further reinforced these findings, demonstrating significant differences between each pair of phyla (Table 1). Differences in morphology and physiology among these major taxonomic groups potentially have had a bearing on their morphospace occupation. This highlights evolutionary divergence, rather than the expected convergence, among red, brown, and green seaweeds.

This finding goes against the established foundations of seaweed biology that posited the functional interchangeability of seaweed phyla^22,38^, as SA:V is strongly correlated with other key functional traits^5,30^. The interchangeability paradigm is so prevalent as to be present in seaweed biology textbooks^59^. Seaweed forms and their associated traits might be more phylogenetically constrained than previously thought^5,6,30,32,45,60^.

Genera (Kruskal–Wallis, chi-squared = 129.74, *p* < 1.39 × e^−8^) and species (Kruskal–Wallis, chi-squared = 131.97, *p* = 0.01053) differed significantly in form between themselves, as expected. However, among the seaweed genera, SA:V variances were homogenous (Levene, F = 1.1487, *p* = 0.2842), highlighting that genera tend to be similar in terms of form variance. In contrast, species SA:V variances are not uniform (F = 5.6475, *p* < 0.001), indicating that species significantly display higher and lesser form variance. A genus of seaweeds, therefore, is expected to have species whose forms vary, yet sum to an equal amount in form variance at the generic level. This might highlight phylogenetic constraints over morphospace exploration, with species occupying areas of different extents, but still circumscribed by genera of overall equal morphospace occupation areas.

The unexpected nature of these results can be understood through taxon cycles. Supposing the incidence of taxon cycle-like mechanisms, a species may vary traits up to a point whereby different niche exploration might lead it to speciate^61–69^, given that polymorphism is unstable in most cases^24,70,71^. Therefore, the trait variance at the species level should not be expected to be homogenous, but the sum of this variation after a cladogenesis event among extant species is expected to result in the conservation of the same variance value at their now genus level. For example, species A has a variance value of 0.5. If speciated into species A’ and A’’, the variance of species A’ and A’’ should necessarily add to 0.5, assuming other parameters remain constant. Species A’ and A’’ would also have heterogenous variances and display different trait values. This is necessarily the case because in a continuous uniform distribution, the probability of a 0.25 and 0.25 division result rounds to zero.

Intrageneric variance (7.75 × 1015, raw data; 0.0138, log-transformed), was consistently lower than intergeneric variance (4.14 × 1016, raw data; 0.737, log-transformed). The F-ratios for these comparisons were 0.0187 (raw data) and 0.0188 (log-transformed), indicating that the variability between genera is substantially higher than within genera. The same mechanism in the illustrative example should apply to explaining this intra- and intergeneric difference, increasing the distribution unevenness proportionally to increase in taxonomic rank (by cladogenesis events). Changes in ecological and physiological dynamics are to be expected, but the pure mathematical explanation of set division is more parsimonious, at least as a proximate causal mechanism.

Conversely, intraspecific variance (4.02 × 10^9^, raw data; 2.86, log-transformed) was higher than interspecific variance (1.19 × 10^5^, raw data; 0.31, log-transformed), with an F-ratio of 9.26. Therefore, variability within species is greater than that between species. This is also to be expected mathematically by the mechanism we illustrated (properties of the uneven division of a set^72^). Regardless, many other effects cannot be excluded and should nonetheless be explored^73^.

### Discrete groups for continuous data: problems in the functional group approach

Categorical groups defined by qualitative morphological traits lay at the foundations of plant and seaweed functional ecology^39^. The seaweed functional groups have been used to differing degrees of success across many applications^43,44,74,75^.

We have demonstrated that seaweed forms are ordered in a continuous and exponential distribution across their geometrically possible morphospace. No significant gaps or clusters were found in the data to support the view that seaweeds cluster towards similar forms thereby entailing similar functional traits. Moreover, 70% of Littler and Littler’s groups and 62% of Steneck and Dethier’s were statistically the same when compared group by group on the account of surface area to volume differences. Therefore, there is no morphological support for current functional groups of seaweeds. Our results also show no support for the claim that seaweeds have been convergently evolving into the same discrete ecomorphs, as assumed by functional group approaches^22,30,38^, since no such form clusters were found. Steneck and Dethier’s proposal was expected to be more accurate than Littler and Littler's due to there being one more group over the latter’s model. As the data on seaweed form has a continuous exponential distribution, any further “categorical” division would render a proposal more accurate, in a way similar to the logic of integral calculus’s infinitesimal treatment. Necessarily, any finite number of functional groups proposed would not adequately fit the distributional data of seaweed form.

The lack of difference between most functional groups of these two models, on which lay the foundations of seaweed functional ecology^59^, puts into question their usefulness and might be the reason behind their very low predictive power over functional traits^6^. These models categorize species based on specimens with a “representative” morphology^23,34^. Morphological change over development and alternation of generations is left unaddressed in all current functional models^30,60^. As a continuous distribution was found, the use of functional group models leads to major information loss. Nonetheless, it could remain useful for applications requiring a lower accuracy threshold.

Seaweeds’ individual-, space-, and time-contingent form variation can be captured by surface area to volume ratio (SA:V) measurement. Due to the strong correlations reported in the literature of SA:V and key functional traits, a numerical and continuous SA:V-based functional model could potentially improve our current trait prediction capability perhaps even on a per-individual basis^76^, shifting the paradigm from group-average qualitative assessment^6,22,38^ towards per-individual quantitative SA:V-based model. Determining whether such a model could directly result in greater simplicity and/or predictive power is valuable topic for future research.

## Conclusion

### The dawn of a new paradigm in seaweed biology?

The functional group approach has led to major advancements in seaweed biology for over 49 years^30,77^, but it is time to move beyond the qualitative and categorical paradigm^5,45^. We showed that foundational assumptions in seaweed biology were not supported by meta-analytical data on seaweeds from around the world. We found that the current seaweed functional models, being predicated on a discrete and categorical distribution of seaweed forms, have very low accuracy in grouping seaweeds by their morphology. We hypothesize that this might be the culprit behind their overall very low predictive power regarding other traits and community processes^5,6,30^. We propose that seaweed functional ecology should strive towards quantitative and continuous approaches that use the seaweed surface area to volume ratio (SA:V) as a key trait for the strong correlations we found in the literature regarding other algal and reef community traits. Additionally, SA:V is simple and easy to measure with accuracy and precision even by non-specialists, whereas traditional functional models often are subjective and require expert knowledge^5,43^. New functional models predicated on seaweed SA:V have the potential to make seaweed biology more quantitative and predictive, thereby improving coastal monitoring, and revolutionizing seaweed farming and industry^78^.

## Supplementary Information

Supplementary Information 1.

Supplementary Information 2.

Supplementary Information 3.

## Data Availability

All data supporting the findings of this study are available within the paper and its Supplementary Materials (1–3).
