# Supplementary material for: The distribution of seaweed forms and foundational assumptions in seaweed biology
Source: Sci Rep. 2024 Sep 28;14:22407. doi: 10.1038/s41598-024-73857-z (PMC11436886; doi:10.1038/s41598-024-73857-z)
Supplement: Supplementary file 1 — Supplementary Information 1. [file 41598_2024_73857_MOESM1_ESM.pdf]

PRISMA 2020 flow diagram for new systematic reviews which included searches of databases and registers only

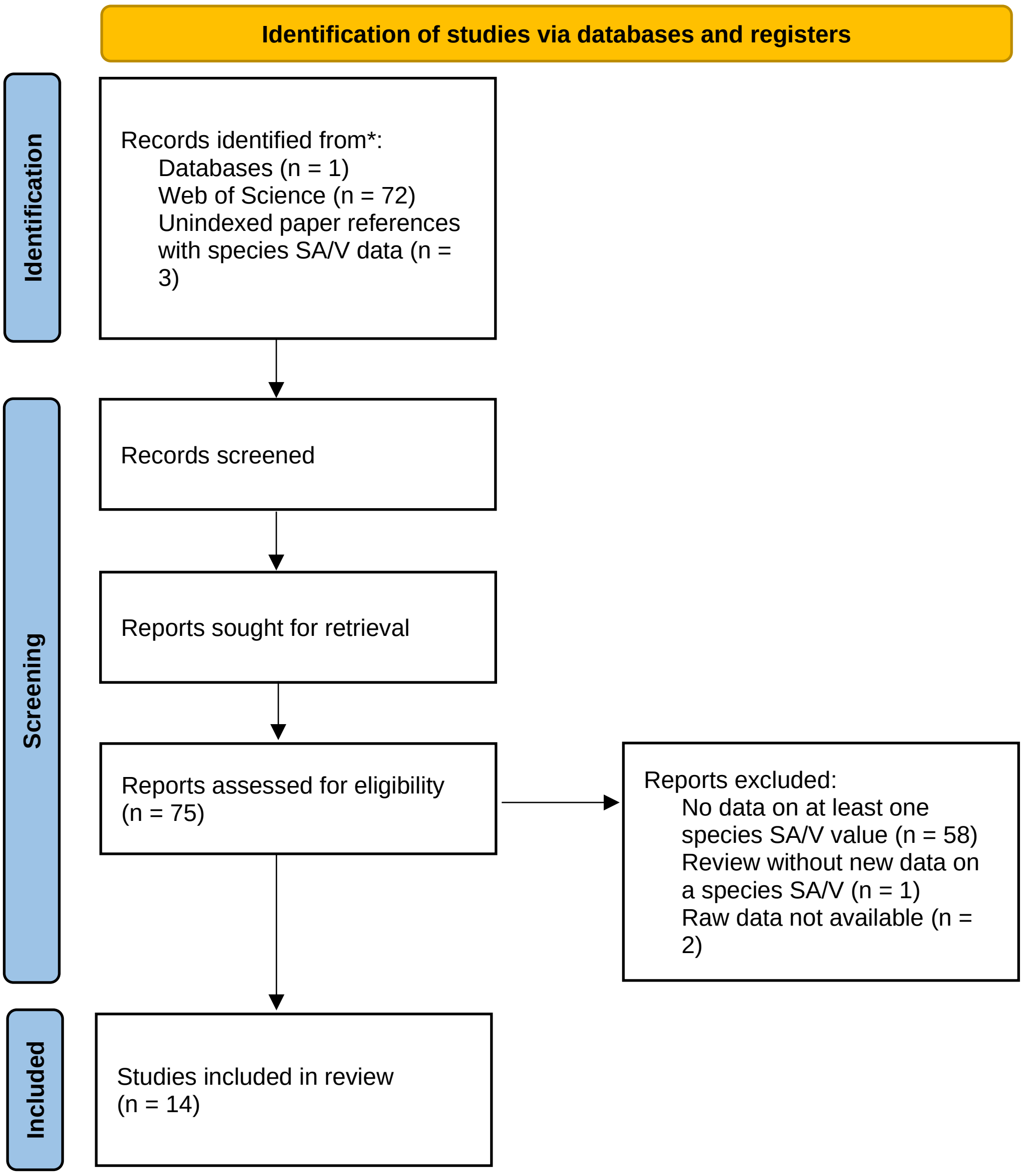

From: Page MJ, McKenzie JE, Bossuyt PM, Boutron I, Hoffmann TC, Mulrow CD, et al. The PRISMA 2020 statement: an updated guideline for reporting systematic reviews. BMJ 2021;372:n71. doi: 10.1136/bmj.n71

For more information, visit: http://www.prisma-statement.org/
